# The Development of Nonthermal Plasma and Tirapazamine as a Novel Combination Therapy to Treat Melanoma In Situ

**DOI:** 10.3390/cells12162113

**Published:** 2023-08-21

**Authors:** Matthew Yehl, Dominik Kucharski, Michelle Eubank, Brandon Gulledge, Gamal Rayan, Md Gias Uddin, Genevieve Remmers, Eugene S. Kandel, Douglas P. DuFaux, Timothy C. Hutcherson, Sandra Sexton, Shoshanna N. Zucker

**Affiliations:** 1 D’Youville University School of Pharmacy, 320 Porter Avenue, Buffalo, NY 14201, USA; 2 IDEXX Laboratories, 1 IDEXX Drive, Westbrook, ME 04092, USA; 3 Roswell Park Comprehensive Cancer Center, 665 Elm Street, Buffalo, NY 14203, USA; 4 Alfie Technology Corporation, 227 Thorn Avenue, Orchard Park, NY 14127, USA

**Keywords:** nonthermal plasma, tirapazamine, medical device, melanoma, skin cancer, gap junctions, porcine skin

## Abstract

Although melanoma accounts for only 5.3% of skin cancer, it results in >75% of skin-cancer-related deaths. To avoid disfiguring surgeries on the head and neck associated with surgical excision, there is a clear unmet need for other strategies to selectively remove cutaneous melanoma lesions. Mohs surgery is the current treatment for cutaneous melanoma lesions and squamous and basal cell carcinoma. While Mohs surgery is an effective way to remove melanomas in situ, normal tissue is also excised to achieve histologically negative margins. This paper describes a novel combination therapy of nonthermal plasma (NTP) which emits a multitude of reactive oxygen species (ROS) and the injection of a pharmaceutical agent. We have shown that the effects of NTP are augmented by the DNA-damaging prodrug, tirapazamine (TPZ), which becomes a free radical only in conditions of hypoxemia, which is often enhanced in the tumor microenvironment. In this study, we demonstrate the efficacy of the combination therapy through experiments with B16-F10 and 1205 Lu metastatic melanoma cells both in vitro and in vivo. We also show the safety parameters of the therapy with no significant effects of the therapy when applied to porcine skin. We show the need for the intratumor delivery of TPZ in combination with the surface treatment of NTP and present a model of a medical device to deliver this combination therapy. The importance of functional gap junctions is indicated as a mechanism to promote the therapeutic effect. Collectively, the data support a novel therapeutic combination to treat melanoma and the development of a medical device to deliver the treatment in situ.

## 1. Introduction

Global cancer data estimated that there were 325,000 new melanoma cases and 57,000 deaths due to melanoma in 2020 [1]. While melanoma accounts for approximately 1% of all skin cancers diagnosed in the United States, it is the deadliest and most aggressive form if not detected early and is responsible for most skin cancer deaths [2,3]. Current standards of care for melanoma include direct applications to the tumors including surgical removal and photodynamic therapy (PTD), as well as systemic treatments including immunotherapy and targeted drug therapy.

While chemotherapy was one of the earliest treatments for advanced melanoma [3], it has no longer been utilized as the first line of treatment because of not being as effective as immunotherapy and targeted drug therapy [4]. Generally, radiation therapy is not widely used to treat melanoma; however, there are cases when it is useful, and those include palliative care, as a supplement to surgery, and when surgery is not an option [5,6].

Surgical treatments of melanoma include either definitive surgical resection which may have remaining positive margins in 6–20% or tumors that can lead to recurrence [7] and Mohs surgery. Mohs surgery involves serial sections of a tumor and examining each under the microscope until the tumor is removed [8]. Although Mohs surgery is effective, normal tissue is also excised, leaving scars that can be disfiguring, particularly on the head and neck region. Average surgical margins of 12 mm are necessary to achieve histologically negative margins in 97% of head and neck melanomas [8,9].

PTD involves the administration of a light sensitive drug known as the photosensitizer to cancer cells followed by its excitation using a laser. The excitation of the photosensitive drug results in the production of reactive oxygen species (ROS), which in turn cause cell and biomacromolecule (i.e., proteins, nucleic acids, and lipids) damage [10]. PTD is more advantageous than conventional treatments such as chemotherapy and radiation therapy, as it is more localized and it produces less destruction; however, it generates heat which can lead to undesirable effects. 

Immunotherapy has demonstrated long-lasting responses to metastatic melanoma, particularly using a combination of checkpoint inhibitors to anti-PD-1 and anti-CTLA-4 antibodies [11]. However, 60–70% of melanoma patients do not respond to checkpoint inhibitor therapy due to toxicity, intrinsic resistance, and other reasons not completely understood, rendering immunotherapy ineffective for them. Moreover, immunotherapies are very expensive and may not be warranted in all situations [12]. 

Targeted drug therapy with medications such as vemurafenib target a BRAF600 mutation that is found in approximately 50% of melanoma patients [13]. This approach is often coupled with MEK inhibitors [14]. Even with combination therapy, there is a high degree of resistance and recurrence, which is often fatal [15]. Like immunotherapy, targeted drug therapies are also very expensive and only effective for some patients [16].

In this paper, we present a combination of nonthermal plasma (NTP) and tirapazamine (TPZ) as a new therapy that shows enhanced therapeutic effects on melanomas. NTP is the production of plasma through the ionization of a neutral gas using an electric field. This application of plasma does not significantly increase the temperature of the areas it is applied to and so will not burn the tissue. NTP exerts its cytotoxic effect primarily through the production of ROS, inducing oxidative stress and DNA damage as a topoisomerase inhibitor, leading to apoptosis in targeted cells [17,18]. ROS production via NTP has a selectively cytotoxic effect within melanoma as compared to normal keratinocytes in vitro [19]. NTP has been demonstrated to have a cytotoxic effect on cancer cells from in vitro and in vivo experiments [20,21,22,23]. More recently, NTP has been utilized in phase I clinical trials which showed successful parameters for safety following surgical resection in solid tumors [24]. NTP has also been shown to induce damage-associated molecular patterns to provoke immunogenic cancer cell death that, in turn, activates the cells of the innate immune system to promote adaptive antitumor immunity [25]. Through an abundance of ROS and reactive nitrogen species delivery to the target tissues, NTP can induce a state of hypoxia through the activation of hypoxia inducible factor 1 (HIF-1) pathways [26]. These results led to the investigation of a combination treatment with a hypoxia-inducing prodrug that has progressed to phase II and III clinical trials, TPZ [27]. TPZ is a prodrug with selectivity towards cancer cells. The selective toxicity of TPZ is attributed to its reduction within a hypoxic environment to its radical anion form, which induces DNA radicals and breakage [28]. The induction of TPZ into its radical form via a hypoxic environment leads to an increase in ROS [29]. Tumors frequently outgrow their blood supply resulting in localized hypoxia of the tumor, which promotes selective cytotoxicity within a solid tumor as compared to healthy tissues.

NTP and TPZ act in concert to deliver ROS to, and induce oxidative stress in, cancer cells. Cancer cells have higher intrinsic levels of ROS and are, therefore, more susceptible to oxidative stress than normal tissues [19,30,31]. It has been established that there is a direct role of ROS in cell damage caused by ultraviolet radiation in melanoma, causing melanocytes to be more susceptible to oxidative stress than keratinocytes and fibroblasts due to melanin production [32]. Ion channels localized to the plasma membrane are also elevated in melanoma, which are targets of oxidative stress [33]. Thus, the targeting of melanoma cells by NTP + TPZ therapy represents a mechanism to induce ROS activation pathways and to selectively induce cell death in melanoma, without damaging normal skin tissue.

In this paper, we confirm the specificity of NTP + TPZ combination therapy by comparing the response in nontumor tissue using porcine skin, which is considered the best substitute for human skin [34,35,36], to the response in tumor tissue using a syngeneic mouse model. Furthermore, we have developed a medical robotic device to administer the therapy which is patent-pending [37]. This novel combination therapy may prove to be a quick, inexpensive, and safe pre-treatment to reduce tumor volume prior to surgery and as a post-surgical adjuvant treatment. This method may also be useful when the primary tumor cannot be surgically removed, i.e., because it is contraindicated, or surgery is refused by the patient. An example of this scenario is lentigo malignant melanoma which may cover very large areas, so that the surgical removal is very disfiguring, or not possible.

## 2. Materials and Methods

### 2.1. Cell Culture and IC_50_ Values

B16-F10 and 1205 Lu cells were maintained in DMEM with 10% FBS and 1% penicillin/streptomycin. Human epidermal keratinocytes were purchased from ATCC and maintained in keratinocyte media (EpiLife media with the addition of Human Keratinocyte Growth Supplement from ThermoFisher Scientific, Grand Island, NY, USA). 1205 Lu P, 1205 Lu C, and 1205 Lu T cells were developed as previously described to express either an empty vector (1205 Lu P), a Connexin 43 (Cx43) construct (1205 Lu C), or a dominant negative Cx43 (1205 Lu T) all linked to GFP through an IRES vector [38]. All cells except keratinocytes were maintained in DMEM with 10% FBS and 1% penicillin/streptomycin. TPZ (SR-4233) was purchased from Sigma Aldrich (St. Louis, MO, USA). IC_50_ for TPZ were compared for B16-F10 cells at normal 21% oxygen (normoxia) versus hypoxia. Cells were plated at 2 × 10^5^ per 96 well and treated with a serial dilution of TPZ. Plates were incubated either at normoxia or in the hypoxic chamber (Biospherix Model^#^E702) within a cell incubator, regulating the oxygen level at 0.1% O_2_. Cell viability was assayed with presto blue (ThermoFisher Scientific, Grand Island, NY, USA). Absorbance at 530 nm was measured using a plate reader (BioTek Synergy HT, Winooski, VA, USA). The data were analyzed with GraphPad Prism version 9.

### 2.2. In Vitro Analysis of Novel Combination Therapy

A comparison of NTP treatment either immersed in cell culture media during treatment (with media during NTP treatment) or when the media was removed just prior to treatment and added back immediately after treatment (without media during NTP treatment) was performed to optimize conditions for therapeutic application. These experiments were conducted to determine the potential benefits of the surface application of NTP directly to the skin in the absence of liquid. B16-F10 cells were plated in 24-well plates and treated with NTP either in the conditions described as with or without cell culture media. The media were removed from the cells just prior to treatment and fresh media were added after treatment with confirmation that no drying out of the cells occurred during the treatment time frame of 30 s. The cells were then incubated with DMEM ± 20 mM TPZ in the hypoxia chamber for 24 h. Cell viability was measured with presto blue (ThermoFisher Scientific, Grand Island, NY, USA) using a plate reader (BioTek Synergy HT). The graph is a representative experiment that was repeated three times. The data were analyzed with GraphPad Prism version 9.

### 2.3. In Vivo Analysis of Novel Combination Therapy

To study the effects of NTP and TPZ on mouse tumors, 20 male and 20 female C57BL/6 mice (10 weeks old) were purchased from the National Cancer Institute (NCI) Mouse Repository (Frederick, MD, USA). These mice were injected intradermally in the right flanks with B16-F10 melanoma cells. The cells (2 × 10^5^ per injection) were delivered in 0.1 mL of PBS. Treatments were initiated when tumors reach 5 mm in their longest dimension (12–14 days post-inoculation). The groups were randomized into four treatment conditions with 10 mice per condition: (1) saline injection, (2) NTP alone, (3) TPZ alone, and (4) NTP + TPZ. The conditions for NTP were two 30 s treatments at 147–200 kHz with a flow rate of 4 L/min at a distance of 1 cm from the source. TPZ was injected at a concentration of 20 μM with a volume of 100 μL using a 28-gauge needle. The concentration of 20 μM TPZ was utilized due to the treatment conditions our previous in vivo studies with metastatic melanoma cells in mice [23]. Five treatments in total were applied to the tumors (every 2–3 days). The mice were examined daily for signs of distress and tumor growth. Tumors were measured in two dimensions using calipers to extrapolate the cubic mm volume. Growth rates were calculated. All mice were euthanized with carbon dioxide at their endpoint according to the IACUC protocol.

Histological analysis of the tumors was performed with hematoxylin and eosin staining. Submitted tissues were trimmed according to the Registry of Industrial Toxicology Animal Data protocols [39], processed for paraffin embedding followed by standard hematoxylin and eosin staining and followed by histopathologic evaluation. The slides were analyzed by a veterinary pathologist.

Microscopic tumor cell death (necrosis) was graded for severity utilizing the following grading system whereby 0 = absence of cell death and necrosis, 1 = less than 25% of cell death and necrosis, 2 = 25% to 50% of cell death and necrosis, 3 = 50% to 75% cell death and necrosis, and 4 = 75% to 100% of cell death and necrosis.

In an experimental analysis of mouse xenografts with 1205 Lu melanoma cells, we performed a direct comparison of intravenous (IV) to intratumor (IT) delivery of 20 mM TPZ to C57BL/6 mice. 1205 Lu C cells were injected subcutaneously through the side flanks (1 × 10^5^ per injection) in 0.1 mL of PBS volume per injection. When the tumors reached 5 mm in their largest dimension, the mice were treated with NTP, TPZ, or NTP + TPZ. The TPZ was either injected through tail vein injection (IV) or delivered directly to the tumor (IT). The tumor volume was followed and measured every two days for a total of ten days and graphed.

### 2.4. Model of the Novel Device to Treat Melanoma In Situ

A device was developed to treat tumors in situ with NTP + TPZ treatment through repeated injection of TPZ with NTP treatment. This rendering of the device can be modified to suit the needs of a physician to treat with the combination therapy in an office setting. The device is patent-pending [37].

### 2.5. Isolation and Treatment of Porcine Skin Punch Biopsies

Freshly frozen porcine skin was obtained from the Comparative Oncology Shared Resource at Roswell Park Comprehensive Cancer Center. Uniform punch biopsies of porcine skin fitting the size of the wells in a 96-well plate were inserted into 96-well plates. Six replicates per condition were utilized and either untreated or treated with NTP for 60 s at 147–200 kHz with a flow rate of 4 L/min at a distance of 1 cm from the source. A total of 100 microliters of DMEM were added to each well with or without 20 mM TPZ. Plates were incubated either at normoxia or in the hypoxic chamber at 0.1% O_2_. After 24 h, the plates were read at 530 nm absorbance with a plate reader. The data was plotted in a boxplot with the standard deviation calculated per condition. 

### 2.6. Experimental Data and Statistics

The F-statistic (F) and the degrees of freedom (df) were calculated and presented in the figure legends. A student *t*-test or a one-way analysis of variance (ANOVA) was used to check statistical significance where applicable. GraphPad Prism version 9 was used to determine the *p* values with * < 0.05, ** < 0.01, *** < 0.001, and **** < 0.0001. ns indicates no significance.

## 3. Results

**Figure 1:**
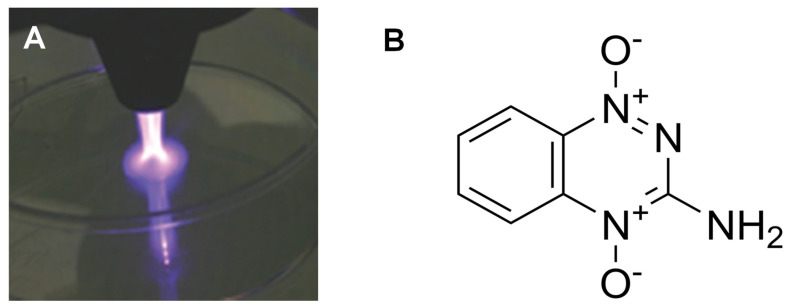
This is an image of the beam of nonthermal plasma emanating from the torch head and targeting a tissue culture dish (**A**). This is the structure of tirapazamine in its active state with the hydroxyl group exchanged for an oxygen radical (**B**).

The NTP and TPZ therapeutic conditions were optimized for melanoma in vivo as a treatment with NTP for 60 s at 147–200 kHz with a flow rate of 4 L/min at a distance of 1 cm from the source [23]. In Figure 1A, a plasma beam emitted from the torch head applied to a tissue culture dish is shown. The structure of the active form of TPZ is shown in Figure 1B. TPZ cycles between its active and inactive forms, activating only in hypoxic conditions [29,40].

### 3.1. In Vitro Results with NTP + TPZ Treatment

**Figure 2:**
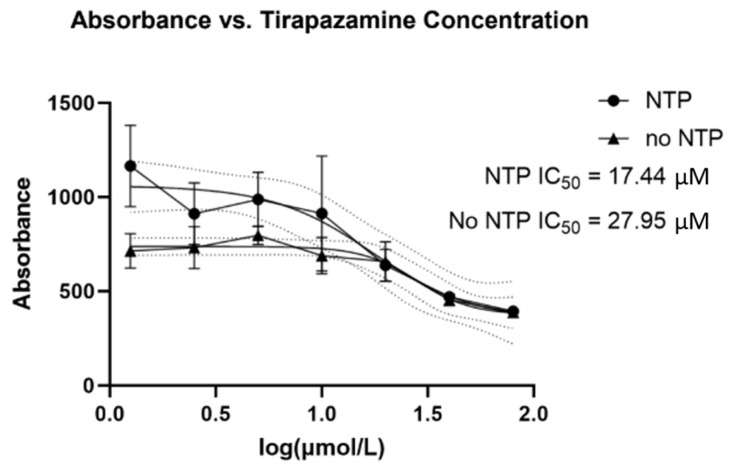
Tirapazamine IC_50_ for B16-F10 cells under hypoxic conditions in the absence or presence of nonthermal plasma.

**Figure 3:**
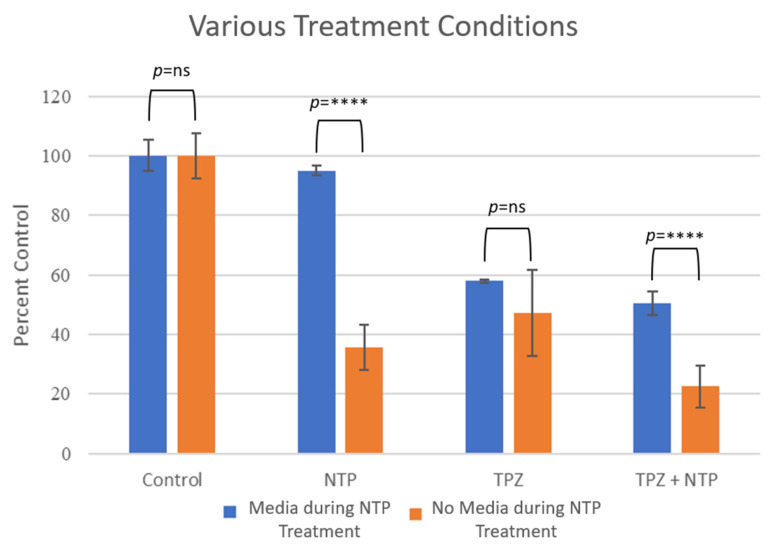
Comparison of murine B16-F10 cells under various treatments when treated with nonthermal plasma in the presence or absence of media. F statistic (in the presence of media during NTP treatment) = 229.9; F statistic (in the absence of media during NTP treatment) = 121.6 (total df for both conditions = 11). **** *p* < 0.001.

The IC_50_ for TPZ was determined in the absence or presence of NTP for B16-F10 cells. Without NTP, the IC_50_ was determined to be 27.95 mM which was reduced to 17.44 mM in the combination of treatment with NTP, demonstrating the additional efficacy of the combination therapy (Figure 2). To determine whether the NTP was more effective when treating the B16-F10 cells in vitro in the absence or presence of DMEM media, experiments were conducted with plasma treatment followed by TPZ in the absence or presence of DMEM. The results demonstrated significant effects only in the absence of media during the treatment time (Figure 3). The experiment was repeated three times, showing a representative experiment in Figure 3. Furthermore, the *p*-value with media during treatment between NTP and NTP + TPZ is <0.0001 (****). Without media during treatment, the *p*-value between NTP and NTP + TPZ is 0.0009 (***). This is significant because it demonstrates that the combination therapy is the preferential mode of application of NTP to the surface of the skin.

### 3.2. Syngeneic Mouse Model for NTP + TPZ Treatment

**Figure 4:**
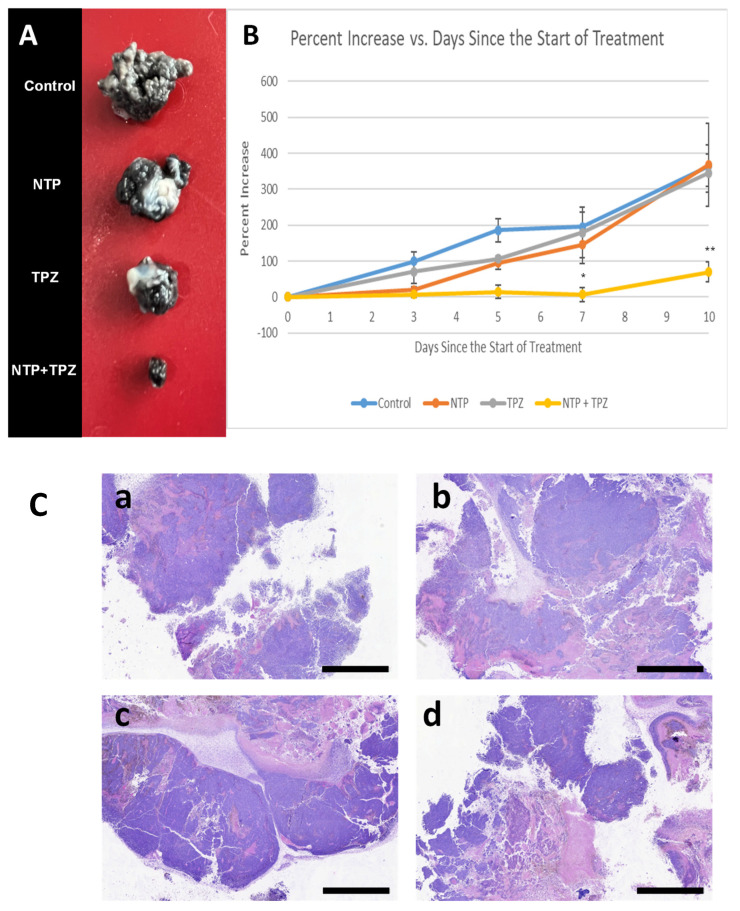
Sample tumors removed from mice post-treatment at day 10 of the experiment at various conditions (**A**). Comparison of the growth rate of tumors at various conditions plotted as the percent increase over time *p* values for * < 0.05, ** < 0.01 (**B**). Histological analysis of tumor sections derived from tumors that were control (**C.a**), treated with nonthermal plasma (**C.b**), treated with tirapazamine (**C.c**), and treated with both nonthermal plasma and tirapazamine (**C.d**). The bars in the images = 2 mm in length.

In these experiments, the tumor volume significantly decreased only in the treatment with the combination of NTP + TPZ (samples of tumor volumes are shown in Figure 4B). The tumor volume was determined by measuring the X and Y axis and applying the formula V = 4π(L1 × L22)/3. The third dimension was difficult to determine since the tumors tended to flatten out as the treatments with the NTP + TPZ progressed. Thus, the growth rate of the tumors was a more accurate depiction of the tumor burden over time (Figure 4B). Moreover, the histological analysis revealed more dead tissue in the tumors from the dual therapy (Figure 4C).

The melanocytic tumor was similar for all conditions. The tumor was characterized by plump to spindled cells forming packets, short streams, and bundles supported by a fine fibrovascular stroma. Neoplastic cells were distinctively bordered with a moderate amount of eosinophilic cytoplasm. The nuclei had finely stippled chromatin and one to three large prominent nucleoli. There was marked anisocytosis and anisokaryosis. Approximately 10–15% of the neoplastic cells contained a small-to-moderate amount of cytoplasmic brownish pigment interpreted to be melanin. There was no definitive evidence of lymphatic or vascular invasion.

The neoplastic cells were associated with variable degrees of randomly distributed areas of cell death and necrosis. The cell death and necrotic areas were characterized by loss of nuclear and cellular details, hypereosinophilic cytoplasm with either complete lack of nuclear details, or deeply basophilic pyknotic nuclei.

Tumor cell death and necrosis were more severe in NTP + TPZ-treated animals compared to control, NTP-, and TPZ-treated mice. The percentage of necrosis of the NTP + TPZ-treated animals was approximately 40%, whereas the percentage of necrosis for all other animals was estimated to be 25%. Thus, the effects of the combination therapy to inhibit tumor growth are likely to be more significant than that represented by panels Figure 4A or Figure 4B. There did not seem to be a difference in percentage of necrosis between the NPT, TPZ, and control samples.

### 3.3. Intravenous Versus Intratumor Administration of TPZ in a Mouse Model

**Figure 5:**
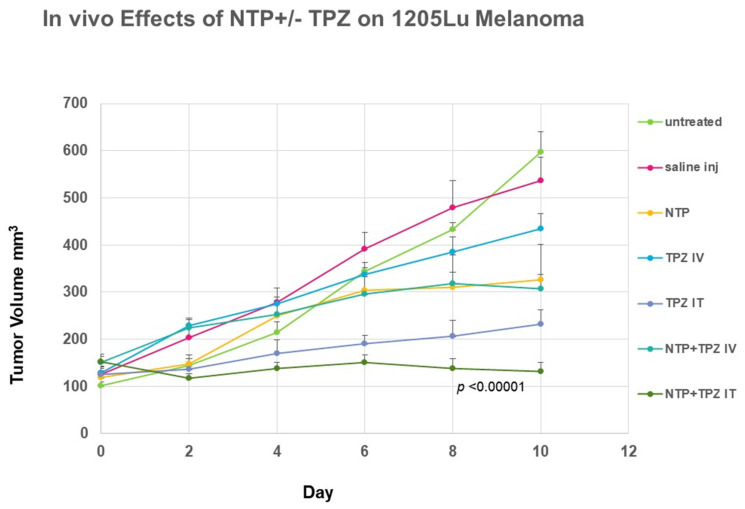
Comparison of the effects of nonthermal plasma and tirapazamine when tirapazamine was delivered intraveneously versus intraturmorally in a mouse xenograft melanoma model using 1205 Lu human melanoma cells.

In a separate analysis of the best mode of delivery for the TPZ, a comparison of intravenous delivery through tail vein injection was compared to intratumor delivery through injection directly into the tumor. These conditions were compared in the presence of absence of NTP treatment. The results for the tumor volume indicated that only the intratumor delivery significantly affected the tumor volume beyond the effect of NTP alone (Figure 5). The intratumor delivery of TPZ is significant as an independent treatment and highly significant as a combination therapy. By day 10 of the tumor growth measurements, the comparison of TPZ intratumor delivery in combination with NTP to the saline treated controls is extremely significant at *p* < 0.00001.

### 3.4. Development of a Medical Device for the Intratumor Delivery of NTP + TPZ

**Figure 6:**
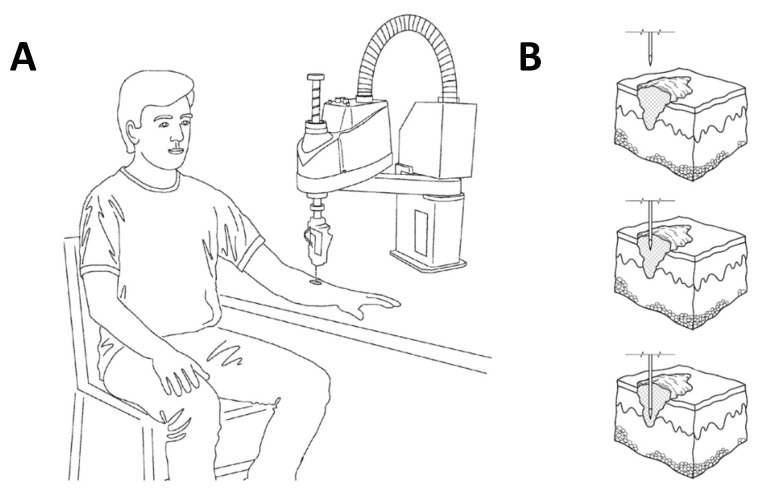
Perspective view of a patient with a melanoma tumor in position to receive a combination plasma–injection therapy provided by a model of our novel medical device (**A**). Model of injection of a needle into a melanoma tumor at increasing depths (**B**).

Due to the significance of the combination therapy, a medical device was created to deliver the combination therapy to a patient. A model of the delivery device is shown in Figure 6A. The combination therapy requires treatment with an injected liquid drug and surface contact of a gas-phase plasma. The liquid drug is delivered through a needle simultaneously with a flow of low temperature plasma stream that impinges the needle and the treatment area. Although treatment can be performed manually, areas larger than a square centimeter benefit from numerous injection sites with small quantities of liquid drug injected at pre-determined depths below the skin’s surface and require an automated delivery system.

The patent-pending automated system [37] is based on a medical-grade multi-axis robot with sufficient degrees of operation to meet the treatment profile developed by a trained clinician. The robotic head can accommodate any contour found on the body to engage the needle with maximum comfort. Treatment is initiated after the trained clinician defines the boundaries of the treatment area and sets the depth and injection volumes and approves the injection site plan determined by the Epson RC version 7.3 controlling software. The robot then begins to articulate and moves to position the head and needle to the first injection position, where the needle is moved to the pre-determined depth and liquid drug is injected, while the plasma gas is continuously flowing over the needle and treatment site.

Figure 6B shows a cross-sectional view of the human skin presenting with a melanoma tumor. The lesion is the portion of melanoma tumor that is visible on skin surface and the melanoma tumor can reach varying degrees of depth, with the inset showing a tumor that has not reached the deepest layer of the skin, the hypodermis. The insets illustrate the impinging treatment needle at a single injection site. The top inset shows the needle approaching the surface of the tumor; the middle inset showing the needle penetrating the surface of the tumor moving into position to administer a cancer treatment drug; and the bottom inset shows the drug delivery needle further embedded within the tumor, at the point where the drug will be administered.

Various versions of the automated robotic system are envisioned for different treatment needs. Compact, portable units using lecture bottle gas supplies and limited articulation range can be used in a physician’s office and within remote institutions. Larger systems with complex robotics can be installed within a hospital or other facility to treat complex tumors, as well as other types of cancers that require deep injections and many individual injections. As the technology progresses, we expect several different types of injectable drugs and plasmas to become available to treat many types of cancers.

### 3.5. Assessment of Safety of the Therapeutic Treatment of NTP + TPZ in Porcine Skin

**Figure 7:**
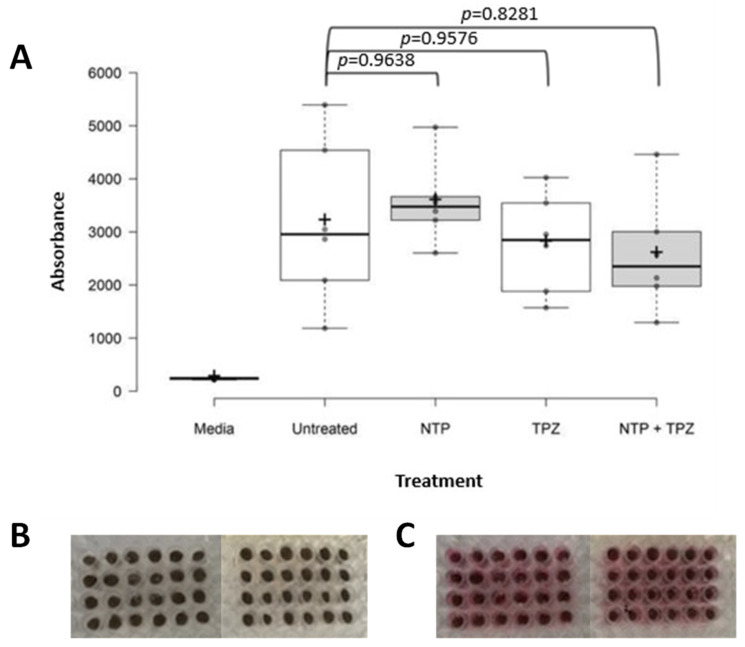
Box plot graph of conditions for treating porcine skin either untreated or treated with nonthermal plasma, tirapazamine, or nonthermal plasma and tirapazamine (**A**). Punch biopsies of porcine skin in 96-well plates prior to treatment in replicates of six per condition (**B**) and after 24 h treatment at hypoxic conditions in media (**C**). F-Statistic = 10.03 (total df = 29) *p* = ns for all conditions.

To determine the safety of the combination therapy on skin, porcine skin was utilized because it is the closest in structure to human skin [34,35,36]. The skin of pigs is composed of an epidermis and dermis with characteristics like those of human skin [36]. Punch biopsies of pig skin of equivalent diameter and thickness were inserted into wells in a 96-well plate. The NTP treatment was applied, followed by media addition in the presence or absence of TPZ. Note that there was no significant effect on viability of the tissue under any of the conditions (Figure 7). (See Section 2 for the statistical analysis.) Media vs. Untreated: 0.0003, ***, Media vs. NTP: <0.0001, ****, Media vs. TPZ: 0.0016, **, Media vs. NTP + TPZ: 0.0041, **, Untreated vs. NTP: 0.9638, ns, Untreated vs. TPZ: 0.9576, ns, Untreated vs. NTP + TPZ: 0.8281, ns, NTP vs. TPZ: 0.6681, ns, NTP vs. NTP + TPZ: 0.4465, ns, and TPZ vs. NTP + TPZ: 0.9959, ns.

### 3.6. Comparison of the Effects of NTP + TPZ on Keratinocytes to Melanoma Cells in the Presence or Absence of Gap Junctional Communication

**Figure 8:**
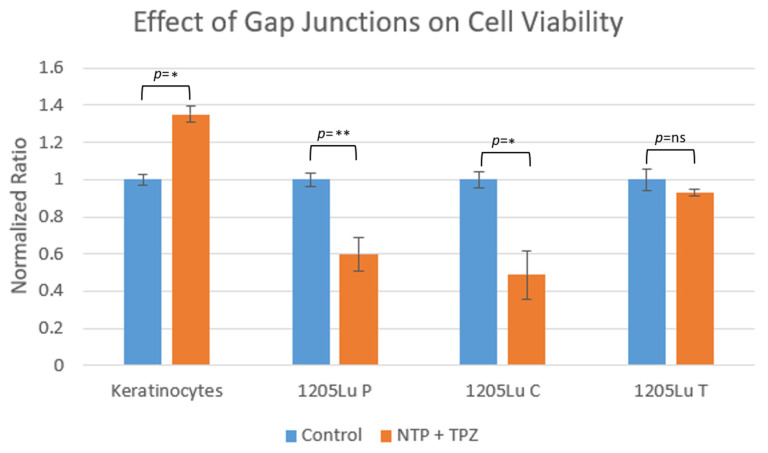
Comparison of viability of keratinocytes and 1205 Lu melanoma cells expressing endogenous gap junctions (1205 Lu P), overexpressed functional gap junctions (1205 Lu C), or dominant negative gap junctions (1205 Lu T) post-treatment with nonthermal plasma and tirapazamine. * *p* < 0.05; ** *p* < 0.01.

To further assess the safety of the therapeutic response of NTP + TPZ, normal human keratinocytes were treated in culture and compared to human melanoma cells either expressing or not expressing functional gap junctions. The graph in Figure 8 shows the difference between the effects of keratinocytes and melanoma cells expressing endogenous gap junctions (1205 Lu P), overexpressed gap junctions (1205 Lu C), and dominant negative gap junctions (1205 Lu T) [23,38,41]. The results demonstrate growth of wild-type keratinocytes, a decrease in growth with 1205 Lu P and 1205 Lu C, but no effect with the cells expressing a dominant negative Cx43 (1205 Lu T). This highlights the effect of gap junctions that has previously been reported [23].

## 4. Discussion

Although significant progress has been made recently in the treatment of melanoma, there are cases where the current standard of care may not be effective. Thus, we have developed a novel ROS-inducing treatment for melanoma in situ. In these experiments, the NTP treatment was assessed either in the presence or absence of cell culture media. The results demonstrated that NTP treatment was only significantly effective in melanoma cells when the cell culture media was removed prior to treatment. This is significant because it demonstrates the ability of our treatment to be effective through intratumor delivery on the surface of the skin.

In our in vivo model, using the syngeneic mouse model, the growth rate of B16-F10 metastatic melanoma cells was significantly decreased only with the combination of NTP + TPZ, showing synergistic effects. The report from the veterinary pathologist confirmed that: (1) The most significant difference between the treated and control samples was the percentage of cell death and necrosis. (2) Tumor cell death and necrosis were more severe in NTP + TPZ treated compared to control, NTP, and TPZ treated mice. (3) There was no significant difference in percentage of necrosis between the NTP, TPZ, and control samples.

This further demonstrates the benefits of the combination therapy which has previously been determined to be effective in a mouse xenograft model [23]. The mode of delivery was tested by comparing intravenous to intratumor delivery. Note that there was no significant increase in the NTP treatment with intravenous TPZ delivery. The enhanced effect of TPZ was only demonstrated with intratumor delivery. This finding led to the development of an intratumor delivery device which is patent-pending [37].

To assess the safety of the combination therapy for clinical use, porcine skin was utilized. Porcine skin is the closest model to human skin available [34,35,36] and fresh-frozen samples were available through the Comparative Oncology Shared Resource at Roswell Park Comprehensive Cancer Center. When isolated porcine skin was treated at hypoxic conditions with either NTP, TPZ, or NTP + TPZ, there were no significant effects on cell death.

The safety of the combination therapy was further assessed through the comparison of the treatment on normal human keratinocytes versus metastatic melanoma cells. The keratinocytes continued to proliferate despite the treatment with NTP + TPZ. The viability of the metastatic melanoma cells was decreased only in the presence of functional gap junctions, (1205 Lu P and 1205 Lu C), but there was no effect in the presence of the dominant negative gap junctions (1205 Lu T). This provides further evidence for the mechanism that has previously been proposed whereby the bystander effect is necessary to promote the passage of derivatives of the ROS molecules produced by the NTP + TPZ therapy [23]. This mechanism suggests that a further development of the bystander effect may be warranted to promote the functionality of the NTP + TPZ therapy in a clinical trial.

Thus far, cold plasma has been assessed in preclinical treatments for skin cancer showing a reduction in cell proliferation, adhesion, and migration, and an induction of selective apoptosis of neoplastic cells without damaging normal cells [42]. Subcutaneous models provided the earliest and the most promising approach as an anti-tumor modality using cold atmospheric plasma (CAP), demonstrating a drastic tumor volume reduction of more than 50% and significantly extending the life span of mice [43,44]. In another study, it was found that a nanosecond-pulsed dielectric barrier discharge (nsP DBD) completely eradicated a xenografted melanoma tumor in mice after direct treatment on the skin; furthermore, the survival rate of mice increased from 0% to 66.7% 20–40 days after the nsp DBD treatment [45]. Moreover, a fractionated, multi-time consecutive treatment was shown to generate a much better therapeutic effect—such as an immune response—than a single treatment, which was considered to be due to the long-term anti-tumor effect of CAP treatment [44].

TPZ has been tested in lung, cervical, and head and neck cancers in combination with chemotherapy or chemoradiation. Although a phase II trial showed significant clinical outcomes, a phase III trial was essentially unsuccessful due mainly to inadequate hypoxia within the tumors [46]; but, under hypoxic and normoxic conditions, various DNA-repair-deficient cell lines showed significant sensitivity to TPZ [47]. In preclinical studies, TPZ effectively inhibited tumor colony forming in vitro, especially in hypoxic cells and induced cell cycle arrest and apoptosis, as well as downregulating HIF-1a, CA-IX, and VEGF expression [40]. Based on our evidence in this manuscript, we expect a better clinical prognosis with the combination treatment of NTP + TPZ than either agent alone. In addition, the novel therapy can be delivered intradermally through a robotic medical device which is patent-pending.

While previous reports demonstrated that the individual therapeutic application of NTP or TPZ had some success preclinically and clinically, in our experimental analysis, the two therapies work together synergistically to promote extensive reduction in tumor volume. Based on our evidence in this manuscript, we expect a better clinical prognosis with the combination treatment of NTP + TPZ than either agent alone. Intravenous TPZ might fail for the obvious reason: a tumor is hypoxic because blood does not reach it, but if blood does not reach it, then intravenous TPZ will not reach it as well. Therefore, intravenous TPZ cannot reach the very tumors that it should be most effective against. Direct injection into the tumors solves this problem. To further advance the potential for therapeutic delivery to patients, the novel therapy can be delivered intradermally through a robotic medical device which is patent-pending.

The novel medical device is an automated positioning device for administering a low-temperature plasma and drug combination to a patient at a selected site of treatment on the patient as shown in Figure 6A. Plasma itself has unique properties that make it therapeutically valuable. To form plasma, an electric field is applied to a region of gas that strips electrons off the gas causing a breakdown of that gas. The resulting free electrons are accelerated by the electrical field, causing these electrons to not be in a state of thermodynamic equilibrium. The gas field that dominates is at room temperature while the electrons are at an elevated temperature. This temperature difference creates a reactive environment and the reactions of electrons and ions created from the background gas result in the formation of metastable particles, reactive species, radicals, and radiation. The process ultimately achieves an otherwise impossibly dry, chemically reactive environment at room temperature [37].

Transdermal drug delivery has a plethora of advantages over traditional methods of drug administration. Specifically, this delivery method can localize in a non-invasive way and allows for the controlled and sustained release of a selected drug or molecule. Secondly, transdermal delivery avoids first pass metabolism that reduces the concentration of a drug before it can be absorbed by the circulatory system. Currently, the main restriction that transdermal drug delivery encounters relates to the permeability of the skin. Low-temperature plasma enables our device to enable the transdermal delivery of significantly larger drugs or molecules without damage at deeper layers of the skin.

In addition to a manual delivery device, our team has further developed an innovative robotic device, one that only requires inputting a set of key variables into computer software to enable the complete automated delivery of a full plasma and drug combination to predesignated sites on a patient. This mechanism promotes consistent treatment results, eliminating the need for manual adjustments, and can be expected to increase the overall efficacy and efficiency of the combination therapy.

## 5. Conclusions

In this study, we demonstrate through in vitro and in vivo experimentation the benefits of a novel potential treatment for melanoma in situ. Both NTP and TPZ have previously demonstrated some preclinical success; however, particularly in our in vivo studies, we show that the combination therapy has a much stronger effect than each independent treatment. Furthermore, we show for the first time that the treatment is only effective via intratumor delivery and not via intravenous delivery. The experiments in which the media were removed prior to NTP application further supports a model for the surface delivery of NTP in the NTP/TPZ combination therapy. The design of our patent-pending robotic medical device is presented to deliver the surface NTP with the injectable TPZ therapy in situ. This device is designed to be portable, affordable, and able to be administered in a physician’s office. The safety parameters of the NTP/TPZ therapy were explored through treatment on porcine skin, which is the closest model for human skin [34,35,36]. Our experiments demonstrated no significant effect on the viability of the porcine skin post-therapeutic treatment, suggesting selectivity for tumor tissue. This demonstrates that the treatment with NTP/TPZ has the potential to be explored in clinical trials with a possibility to become a novel in situ treatment for melanoma.

## Data Availability

Data available upon request.
